# Evaluation of angulation and distance deviation for robot-guided laser osteotomy – a follow-up study on digital high-tech procedures

**DOI:** 10.3389/frobt.2025.1559483

**Published:** 2025-04-16

**Authors:** Bilal Msallem, Lara Veronesi, Florian Samuel Halbeisen, Michel Beyer, Adrian Dragu, Florian Markus Thieringer

**Affiliations:** ^1^ UniversityCenter for Orthopedics, Trauma and Plastic Surgery, Faculty of Medicine and University Hospital Carl Gustav Carus, TU Dresden, Dresden, Germany; ^2^ Medical Additive Manufacturing Research Group (Swiss MAM), Department of Biomedical Engineering, University of Basel, Allschwil, Switzerland; ^3^ Clinic of Oral and Cranio-Maxillofacial Surgery, University Hospital Basel, Basel, Switzerland; ^4^ Surgical Outcome Research Center, University Hospital Basel and University of Basel, Basel, Switzerland

**Keywords:** 3D printing, dimensional measurement accuracy, laser ablation, mandibular osteotomy, precision medicine, robotic surgical procedures

## Abstract

**Background and objective:**

Conventional osteotomy tools, including drills and saws, have been associated with several limitations, such as restricted cutting geometry and the risk of heat-induced necrosis, which affects bone healing. Laser-based osteotomy systems have emerged as a promising solution for these constraints. This study aims to evaluate the accuracy of robot-guided laser osteotomy compared to conventional cutting-guided osteotomy based on surface scanning.

**Materials and methods:**

Ten 3D printed mandibular models were used to perform segmentectomy. Five models were treated with conventional osteotomies employing a cutting-guided saw technique, while the remaining five were subjected to laser osteotomy. Initially conducted using root mean square (RMS) values, the analysis has been expanded to reevaluate the angulation and distance deviation outcomes.

**Results:**

Precision analysis of the upper cutting plane revealed a statistically significant difference in distance deviation between the laser osteotomy group (0.48 mm) and the conventional osteotomy group (0.78 mm). In terms of angulation deviation, the laser osteotomy group exhibited, both in the upper and lower cutting planes, statistically significant results (2.19° and 2.86°) compared to the osteotomy group (5.15° and 8.12°).

**Conclusion:**

Based on the observed angulation and distance deviations, it can be concluded that robot-guided laser systems achieve significantly higher accuracy in osteotomies than conventional cutting-guided systems currently available. Consistent with the findings of a prior study, these results confirm that robot-guided laser osteotomy provides substantial advantages, facilitating the seamless integration of precise virtual preoperative planning with exact execution in the human body.

## 1 Introduction

Most osteotomies are traditionally performed using manually operated instruments such as drills or saws. However, these tools present several challenges, including mechanical and thermal effects on bone structure, resulting in biological disadvantages (Martins et al., 2011)– (Abu-Serriah et al., 2004). The bone structure can be compromised, potentially leading to necrosis and prolonged bone healing (Gabrić et al., 2016), (Panduric et al., 2014), (Kanazirski et al., 2023). Clinical studies demonstrated superior outcomes in terms of minimized facial edema, swelling and pain in the early post-operative period (Blagova et al., 2023), (Stübinger et al., 2009). Additionally, the range of shapes that can be created using those instruments is limited (Augello et al., 2017). Since their effectiveness depends greatly on the surgeon’s skills in handling them, outcomes can vary significantly. Individual cutting guides are frequently employed to enhance precision and ensure adherence to pre-established cutting paths (Zavattero et al., 2019)– (Bernstein et al., 2017).

Laser-based systems have gained attention as innovative tools for bone-cutting techniques. Erbium-doped Yttrium Aluminum Garnet (Er:YAG) lasers have demonstrated considerable suitability for use in osteotomy procedures (Martins et al., 2011), (De Mello et al., 2008)– (Stübinger, 2010). They operate below the critical temperature for bone necrosis, using a water-absorbable wavelength of 2,940 nm. This absorption causes micro-explosions in water-containing areas of the bone, ultimately enabling precise bone cutting (Panduric et al., 2014), (De Mello et al., 2008), (Zeitouni et al., 2017), (Baek et al., 2015), (Baek et al., 2021). Lasers have a narrower cutting width than other instruments, which results in less bone material being lost as debris (Panduric et al., 2014), (Stübinger et al., 2009), (Augello et al., 2017), (Stübinger, 2010), (Baek et al., 2021). Furthermore, these devices possess the advantage of total freedom in geometry, with the capacity to achieve a vast range of cutting shapes, including self-stabilizing cuts (Holzinger et al., 2021; Bernstein et al., 2017; De Mello et al., 2008). Laser-based systems present a non-contact, blood-, heat- and vibration-reduced alternative to current osteotomy techniques (Augello et al., 2017), (Augello et al., 2018), (Baek et al., 2015), (Burgner et al., 2010). Multiple studies have been conducted on bone healing after laser osteotomy, and results have shown that this technique is safe, efficient, and less invasive (Martins et al., 2011), (Stübinger et al., 2009), (Stübinger, 2010).

To further enhance the advantages of laser-based osteotomy, its integration with a robot-guided system has been introduced. Robot-supported systems are increasingly utilized in the medical field, particularly in surgical procedures (Martins et al., 2011), (Godzik et al., 2019), (Köhnke et al., 2024). These systems aim to improve accuracy and eliminate human errors while increasing efficiency (Baek et al., 2015), (Baek et al., 2021). These systems also contribute to decreased intra-operative time and costs, making them an attractive option in modern surgical practices (Godzik et al., 2019). Moreover, the combination of laser-based osteotomy with robot-guided systems enables the adoption of a fully digital workflow. This approach allows for preoperative digital planning and exact intraoperative execution. In addition to reducing operating time, this also results in increased precision, eliminates the need for cutting guides, and allows for *ad hoc* osteotomy adjustments to the osteotomy procedure (Holzinger et al., 2021), (Ebeling et al., 2023), (Ureel et al., 2021), (Köhnke et al., 2024), (Msallem et al., 2024a).

Although the advantages of laser osteotomes are well-documented and their efficacy and feasibility extensively studied, data on robot-assisted systems remains limited, particularly regarding their accuracy. This study builds upon previous research that employed descriptive data analysis to evaluate mean standard deviation, median, minimum, and maximum of root mean square (RMS) values for surface comparisons of reconstructed mandibular models in relation to trueness and precision (Msallem et al., 2024a). However, as the previous study did not yield statistically significant results for RMS values, a new approach was adopted, focusing on parameters of angulation and distance deviation.

The present study evaluates the accuracy of a robot-guided laser osteotome compared to a conventional osteotomy technique utilizing manually operated tools and supportive cutting guides. Trueness and precision were assessed based on angulation and distance deviation from the pre-planned osteotomy path. The objective is to ascertain the most precise osteotomy technique for surgical applications by re-evaluating the accuracy measurements of these technologies that were previously analyzed.

Furthermore, the study highlights the advantages of a fully digital workflow encompassing pre-operative digital planning and precise intraoperative execution.

## 2 Materials and methods

### 2.1 Study protocol

**FIGURE 1:**
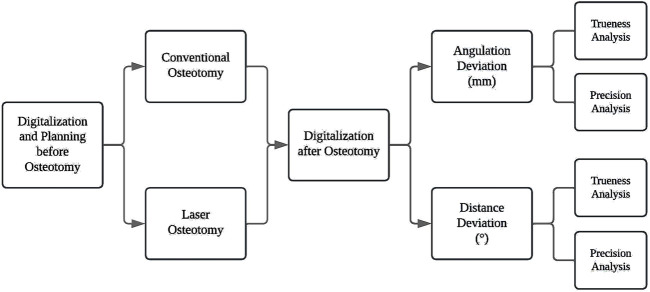
Flowchart of the study protocol.

The following sections outline the study design for evaluating the accuracy of robot-guided laser osteotomy compared to conventional osteotomy using cutting guides. The flowchart visually represents the study protocol, detailing the sequential phases (Figure 1). Ethical approval was not required as this study does not involve animal or human data or tissues.

### 2.2 Digitalization and planning before osteotomy

Ten mandibular models (Models 1–10) were produced for this study using selective laser sintering with an EOSINT P 385 3D printer (EOS GmbH, Krailling, Germany) and white polyamide 12 PA 2200 powder (EOS GmbH, Krailling, Germany). This method was chosen based on its high precision, high accuracy, and the best results in recent studies comparing different printing technologies (Msallem et al., 2020; Msallem et al., 2024b). The mandibular models (Models 1–10) were scanned using a white-light desktop optical 3D scanner (EinScan-SP, SHINING 3D Tech. Co., Ltd., Hangzhou, China). The EinScan-S series software v. 2.7.0.6 was applied to create ten Standard Tessellation Language (STL) files (STL-Planning 1–10). As the printed models were identical, the STL file of Model 1 (STL-Planning 1) was selected for osteotomy planning.

**FIGURE 2:**
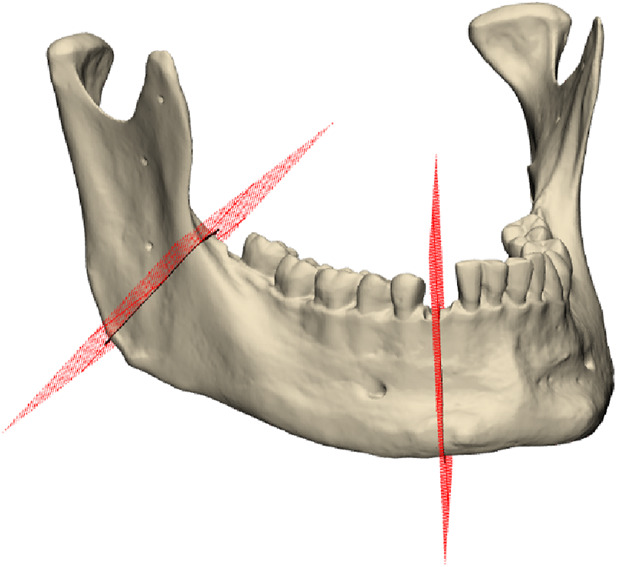
STL-Planning 1 file with the planned cutting planes.

Segmentectomy is defined by two separate cuts on the right side of the mandible: a lower cut is located in the right canine region, while the upper cut is located on the right ascending mandibular ramus, as depicted in Figure 2. Four screw holes accompanied each segmentectomy cut. The planning was conducted in Geomagic Freeform (3D Systems Inc., Rock Hill, SC, United States). Cutting guides for the conventional osteotomy were designed based on the predefined osteotomy lines on the virtual model. These guides included guidance ducts for screw holes as well. An additional positioning aid was incorporated into the incisura semilunaris to facilitate alignment of the cutting line on the ascending ramus. In contrast, the guide for the lower cutting line did not require additional positioning aids, as the uneven contours of the mandibular corpus naturally facilitated alignment.

The cutting guides were 3D printed using dark gray polyamide 12 3D HR (HP Inc., Palo Alto, CA, United States) on a HP Jet Fusion 3D 4200 3D printer (HP Inc., Palo Alto, CA, United States). Following the printing process, the guides underwent sandblasting as a post-processing procedure (DePuy Synthes, Johnson & Johnson, West Chester, PA, United States).

### 2.3 Conventional osteotomy

**FIGURE 3:**
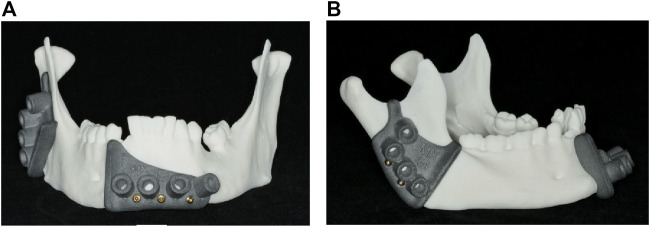
3D printed cutting guides: **(A)** Front view of the lower cutting guide; **(B)** side view of the upper cutting guide.

Models one to five were cut using 3D printed cutting guides, which were positioned on the mandible and fixed with 2.0 mm diameter cortical screws (Medartis AG, Basel, Switzerland), as illustrated in Figure 3. The osteotomy procedure was performed using a Colibri II equipped with an oscillating saw attachment (DePuy Synthes, Johnson & Johnson, West Chester, PA, United States).

### 2.4 Laser osteotomy

**FIGURE 4:**
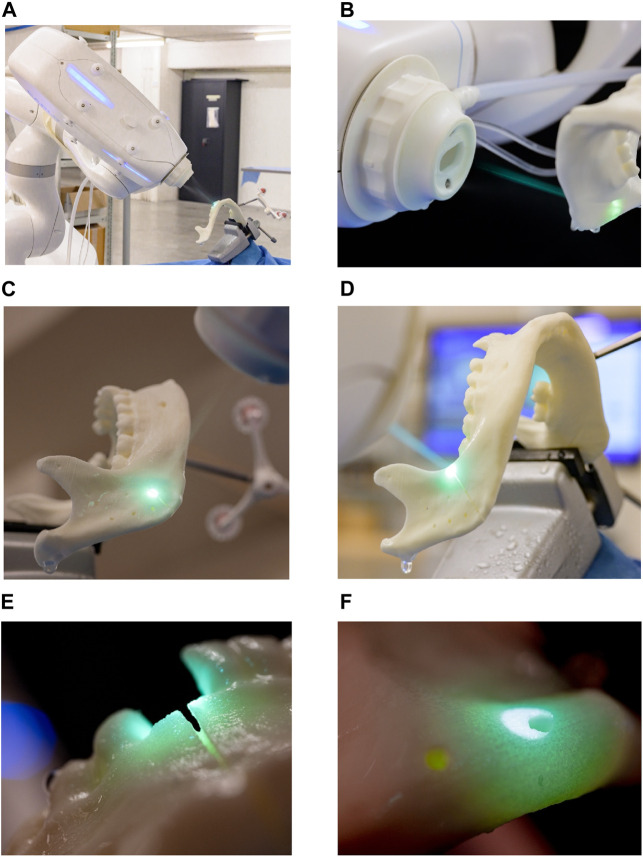
Robot-guided laser osteotomy conducted by CARLO®: **(A)** laser osteotomy set-up **(B–D)** visualization of the cutting process **(E–F)** close-up views of the laser cuts.

The robot-guided laser osteotomy was performed using CARLO® (Cold Ablation Robot-guided Laser Osteotome), an Er:YAG laser device designed for thermal ablation. CARLO® features a scanning laser and an integrated camera for auxiliary visualization (Figure 4). The device has a cutting width of 1.5 mm and a maximum cutting depth of 20 mm. The cutting area is continuously cooled and rinsed with a water spray to maintain precision and prevent thermal damage.

A CARLO® procedure pack (AOT AG, Basel, Switzerland) is required for each surgical intervention, containing disposable materials, such as water tubes for the cooling system. In this study, the same procedure pack was used for all osteotomies. The procedure was executed using the CARLO® primo^+^ software v.2.0.x (AOT AG, Basel, Switzerland). Cutting can be planned using preoperative data, such as a computed tomography (CT) scan or STL files; this study utilized an STL file. Additionally, the system supports *ad hoc* adjustments, allowing modifications to the cutting plan before and during the procedure.

### 2.5 Digitalization after osteotomy

**FIGURE 5:**
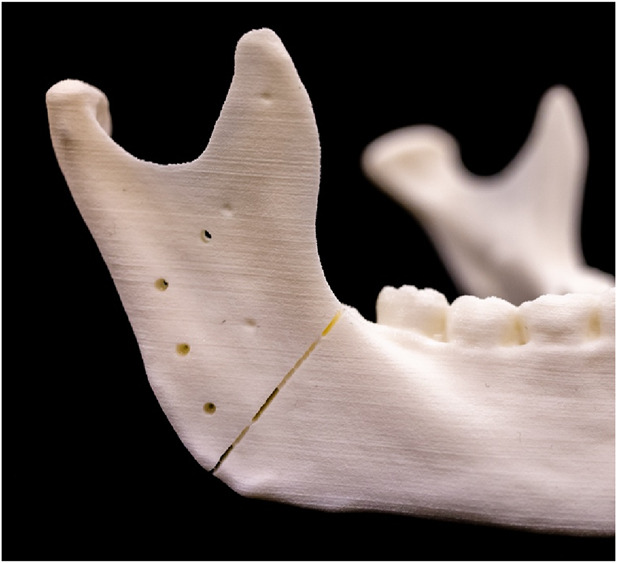
Upper cutting plane with the corresponding screw holes and reference points after laser osteotomy.

To ensure precise scanning of the mandibular models in their entirety, both laser and conventional osteotomies were intentionally designed to avoid cutting entirely through the models. Instead, the incisions were only made through the outer layers of the material, leaving a small core intact to preserve the correct positioning and angulation of the cutting plane, thereby maintaining the stability of the entire model (Figure 5). Following this procedure, the models were rescanned using the previously described digitization method, resulting in a new set of ten STL files (STL-Cutting 1–10).

To evaluate the accuracy of the cuts performed with conventional and laser osteotomies, the STL files were imported into 3-matic medical v. 17.0 (Materialise, NV, Leuven, Belgium) and superimposed onto the planning file. Reference points on the right and left ascending ramus were used, as depicted in Figure 5. Subsequently, a global optimization registration alignment was performed.

For all scanned mandibular models and the planning file, an osteotomy line was digitally inserted at both the lower and upper cutting planes, and the intersection line with the planning mandibular mesh was calculated. Two key assessments were conducted: angulation deviation and distance deviation. Angulation deviation was measured by calculating the angle between the cutting plane of the scanned mandible and that of the planning file for each osteotomy cut. Distance deviation was assessed by calculating the median distance between the scanned mandible’s intersection curve and the planning file’s corresponding curve for each osteotomy cut.

Accuracy in terms of trueness was evaluated by comparing the ten cut mandibles to the planning file. Precision was assessed by comparing the ten cut mandibles within their respective groups: conventional osteotomy models were compared among themselves, and laser osteotomy models were compared within their group.

### 2.6 Statistical analysis

Median values for angulation and distance deviation were analyzed to evaluate the differences between fully robot-guided laser osteotomy and conventional osteotomy. Both trueness and precision were assessed based on deviations in the position of the osteotomy cuts in millimeters and angulation in degrees. All statistical analyses were conducted using the Wilcoxon Rank Sum test, performed with R statistical software (Version 4.3.2, The R Foundation for Statistical Computing, Vienna, Austria).

## 3 Results

### 3.1 Trueness analysis

Table 1 presents the trueness values of the lower and upper cutting lines, separated by the distance and angulation deviation analysis. The values are analyzed for both techniques, focusing on deviations in distance (mm) and angulation (°). Subsequent sections provide a comprehensive examination of these comparisons.

**TABLE 1:** Trueness of distance (mm) and angulation (°) deviation by osteotomy technique.

	Laser osteotomy		Conventional osteotomy		*p*-value
	*n* ^a^	Median (IQR^b^)	*n* ^a^	Median (IQR^b^)	
Distance Deviation (mm) - Lower Cutting Planes	5	0.26 (0.22–0.90)	5	1.09 (0.81–1.14)	0.310
Distance Deviation (mm) - Upper Cutting Planes	5	0.94 (0.53–1.19)	5	0.87 (0.72–1.00)	0.841
Angulation Deviation (°) - Lower Cutting Planes	5	1.86 (1.44–1.91)	5	2.42 (1.97–4.23)	0.310
Angulation Deviation (°) - Upper Cutting Planes	5	2.19 (1.84–2.74)	5	2.44 (1.73–9.97)	0.690

^a^ number of comparisons.

^b^ interquartile range.

#### 3.1.1 Distance deviation trueness

**FIGURE 6:**
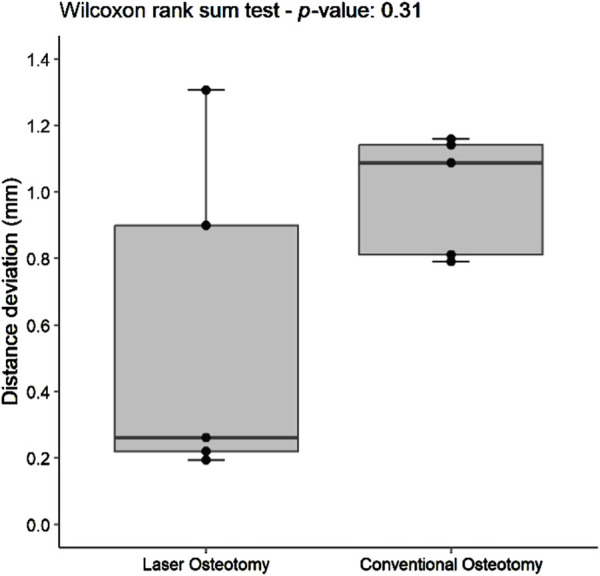
Box plot of the median distance deviation trueness values of the lower cutting plane by osteotomy technique.

**FIGURE 7:**
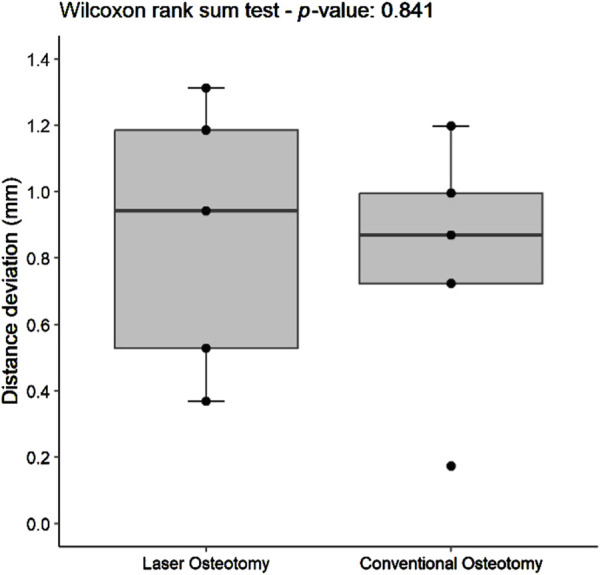
Box plot of the median distance deviation trueness values of the upper cutting plane by osteotomy technique.

Distance deviation was analyzed relative to the planning file, and the median values were calculated for both osteotomy techniques. While differences in median trueness values were observed between the two techniques, no statistically significant differences were identified in the distance deviation for either the lower or upper cutting planes. Nevertheless, laser osteotomy demonstrated superior performance in the lower cutting plane, showing a median distance deviation of 0.26 mm, compared to 1.09 mm for the conventional osteotomy. Conversely, conventional osteotomy exhibited slightly superior outcomes in the upper cutting plane, with a distance deviation of 0.87 mm compared to 0.94 mm for laser osteotomy. These findings are presented in Table 1 and illustrated in Figures 6, 7.

#### 3.1.2 Angulation deviation trueness

**FIGURE 8:**
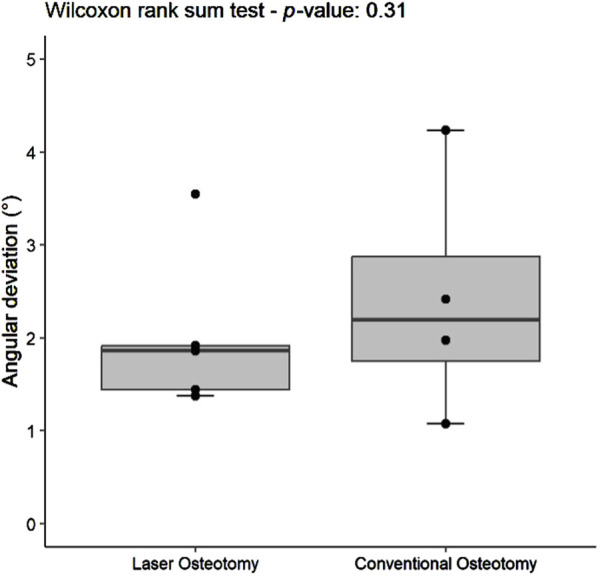
Box plot of the median angulation deviation trueness values of the lower cutting plane by osteotomy technique.

**FIGURE 9:**
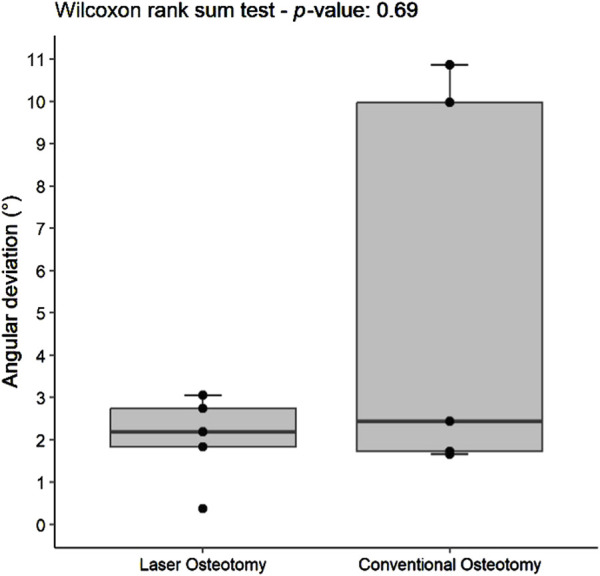
Box plot of the median angulation deviation trueness values of the upper cutting plane by osteotomy technique.

Angulation deviation was assessed relative to the planning file. While differences in median trueness values were observed between the two techniques, no statistically significant differences were found in angulation deviation for either the lower or upper cutting planes. However, the laser osteotomy group exhibited superior performance in the lower cutting plane, with an angulation deviation of 1.86°, compared to 2.42° for the conventional osteotomy group. The laser osteotomy group demonstrated a deviation of 2.19° for the upper cutting plane, compared to 2.44°. These findings are presented in Table 1, highlighting that the laser osteotomy generally exhibited better results, particularly for the lower cutting plane, and are illustrated in Figures 8, 9.

### 3.2 Precision analysis

Table 2 presents the precision values of the lower and upper cutting planes, separated by the analysis of distance (mm) and angulation deviation (°).

**TABLE 2:** Precision of distance (mm) and angulation (°) deviation by osteotomy technique.

	Laser osteotomy		Conventional osteotomy		*p*-value
	*n* ^a^	Median (IQR^b^)	*n* ^a^	Median (IQR^b^)	
Distance Deviation (mm) - Lower Cutting Planes	10	0.66 (0.5–1.08)	10	0.48 (0.35–0.58)	0.143
Distance Deviation (mm) - Upper Cutting Planes	10	0.48 (0.28–0.71)	10	0.78 (0.54–1.58)	0.052
Angulation Deviation (°) - Lower Cutting Planes	10	2.19 (1.03–3.47)	10	5.15 (3.09–6.03)	0.009
Angulation Deviation (°) - Upper Cutting Planes	10	2.86 (1.75–4.39)	10	8.12 (4.66–11.52)	0.009

^a^ number of comparisons.

^b^ interquartile range.

#### 3.2.1 Distance deviation precision

**FIGURE 10:**
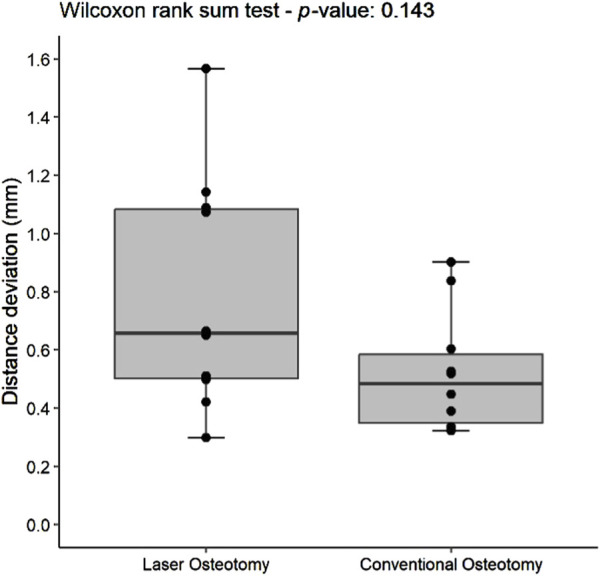
Box plot of the median distance deviation precision values of the lower cutting plane by osteotomy technique.

**FIGURE 11:**
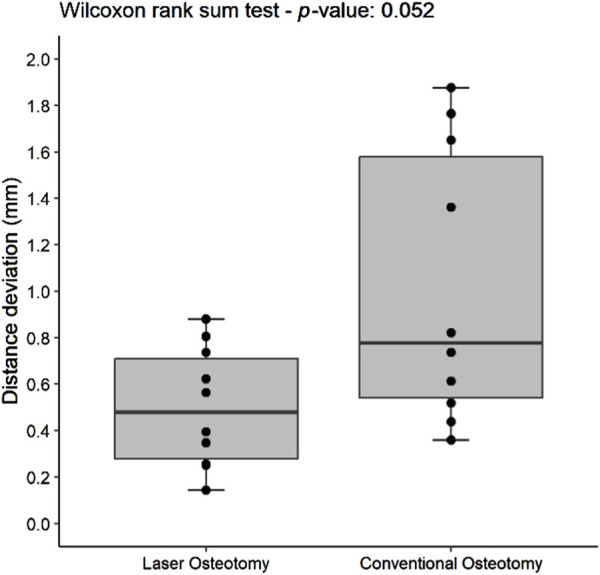
Box plot of the median distance deviation precision values of the upper cutting plane by osteotomy technique.

For the lower cutting plane, no statistically significant difference was observed in the median precision values for distance deviation between the laser osteotomy group of 0.66 mm and the conventional osteotomy group of 0.48 mm. In contrast, a statistically significant difference was identified for the upper cutting plane, with the laser osteotomy group exhibiting a median distance deviation of 0.48 mm compared to 0.78 mm for the conventional osteotomy group. These findings are presented in Table 2 and illustrated in Figures 10, 11.

#### 3.2.2 Angulation deviation precision

**FIGURE 12:**
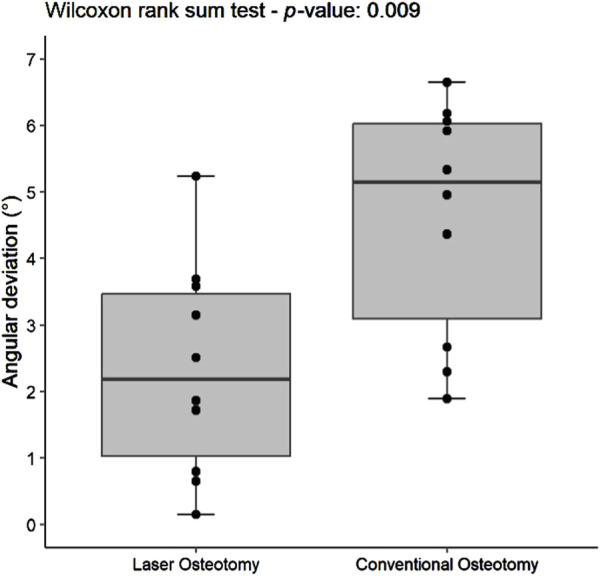
Box plot of the median angulation deviation precision values of the lower cutting plane by osteotomy technique.

**FIGURE 13:**
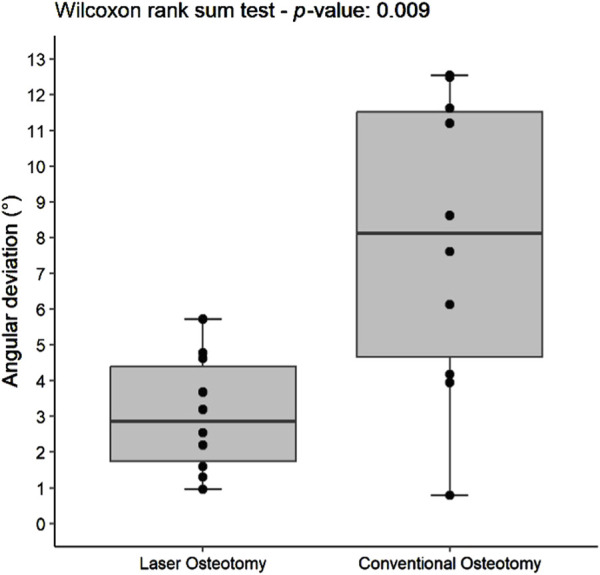
Box plot of the median angulation deviation precision values of the upper cutting plane by osteotomy technique.

The precision values for median angulation deviation of the lower and upper cutting planes differed statistically between the two techniques. For the lower cutting plane, the laser osteotomy demonstrated a median angulation deviation of 2.19°, compared to 5.15° for the conventional osteotomy. Similarly, the median angulation deviation for the upper cutting plane was 2.86° for the laser osteotomy and 8.12° for the conventional osteotomy. These findings are presented in Table 2 and illustrated in Figures 12, 13.

## 4 Discussion

Surgical accuracy is essential, particularly in the anatomically complex region of the facial skull, where it plays a critical role in both functionality and aesthetics (Augello et al., 2018), (Msallem et al., 2024a), (Heufelder et al., 2017), (Han et al., 2021). Robot-assisted systems are increasingly being adopted in medicine for their ability to streamline and optimize surgical procedures. These systems enhance precision and efficiency by incorporating preoperative digital planning and intraoperative navigation. Although robotic guidance has been introduced across various fields, many systems lack full integration for both navigation and surgical execution. For example, robot-assisted pedicle screw placement has demonstrated improved accuracy compared to entirely manual methods. However, while these systems utilize preoperative digital planning and navigation, the surgeon still performs the actual screw drilling (Godzik et al., 2019), (Matur et al., 2023), (MacLean et al., 2024). Similarly, de Boutray et al. employed robot-guided cutting guides to assist with fibular osteotomy, but the surgical procedure was carried out manually (de Boutray et al., 2024). These examples highlight the growing role of robots in medical specialties and underscore the need for continued innovation. The complete integration of robotic systems into surgical workflows is expanding, offering a promising future for the development of fully digitalized and automated surgical systems (Wojcik et al., 2023)– (van Riet et al., 2021).

This study evaluated the accuracy of robot-guided laser osteotomy compared to the conventional cutting-guided saw technique, with accuracy measured in terms of trueness and precision. Distance deviation, defined as the variance between the intended and actual cutting plane, was used to assess positional accuracy, while angulation deviation, defined as the angle between these cutting planes, measured orientation accuracy. These parameters were selected to effectively visualize deviations at different levels, as the precise positioning of cutting planes is crucial for medical decision-making, such as determining the need for subsequent resections.

The results demonstrated statistically significant differences in the precision analysis for the values of the distance deviation for the upper cutting plane and for angulation deviation across both cutting planes in the laser osteotomy group. While no statistically significant differences in trueness analysis were observed, this may be attributed to the smaller group sizes used for trueness (five comparisons per group) compared to the precision analysis (ten comparisons per group). Nevertheless, when absolute figures for angulation deviation in precision and trueness were considered, the laser osteotomy group consistently demonstrated superior performance. However, this was not statistically significant for the trueness analysis. The minor angulation discrepancies at shorter distances result in less significant deviations than those observed at longer distances. This is also reflected in the minimal discrepancy in distance deviation between the two techniques, given that mandibular osteotomies represent relatively short distances. Nevertheless, the robot-guided laser osteotomy achieved superior results across nearly all assessments. These findings underscore the greater accuracy of the robot-guided laser osteotomy technique compared to the current gold standard using prefabricated cutting guides. Additionally, while both osteotomy techniques demonstrated high accuracy, robot-guided osteotomy offers a key advantage: its precise implementation minimizes human and technical errors by eliminating variability in the surgeon’s execution and reducing inaccuracies from interposed production steps. Beyond its superior accuracy, this technique also provides significant biological benefits through laser application and removes the need for cutting-guide production, further enhancing the efficiency of the robot-guided system.

Not only was superior accuracy demonstrated, but biological advantages were also observed–more precisely, the laser osteotome’s ability to operate below critical temperatures. Multiple pre-clinical studies explored the biological advantages of laser-based osteotomy techniques (Panduric et al., 2014), (Kanazirski et al., 2023)– (Abu-Serriah et al., 2004). Naturally, the material’s response to heat differed from that of bone, as bone tends to char while polyamide begins to melt. However, no heat effects were observed with robot-guided laser osteotomy due to its superior biological properties, while conventional osteotomy clearly showed heat effects. While this study focused on laser-based osteotomy compared to the standard saw-based technique, evaluating other osteotomy methods would also be valuable, as each may offer unique benefits when combined with robot guidance.

In the introduction of the aforementioned comparative study, the RMS values were used to assess surface accuracy, as the surface of the entire model was measured to evaluate the accuracy of an anatomical reconstruction, i.e., the clinical endpoint (Msallem et al., 2024a). Consistent with the present findings, that study also demonstrated superior results with higher accuracy in the robot-guided laser osteotomy group. These studies confirm that laser osteotomy offers enhanced cutting precision and improved accuracy in achieving the desired clinical outcomes.

The findings from the present and comparative study, summarized in Tables 3, 4, highlight the advantages of robot-guided laser osteotomy in achieving greater accuracy during cutting and thus improved clinical outcomes. This evidence supports the potential for laser osteotomy to outperform conventional techniques in terms of surgical accuracy and clinical efficacy. Additionally, our previous study on robot-guided laser osteotomy, focusing on the accuracy of subsequent reconstruction after osteotomy, showed no statistically significant differences, despite mainly superior results for the laser osteotomy group. Nevertheless, in both studies, not all cuts demonstrated superior accuracy through robot guidance. However, no trials were excluded from our analysis.

**TABLE 3:** Comparison of the trueness analysis by osteotomy techniques of the present and comparative study.

	Laser osteotomy		Conventional osteotomy		*p*-value
	*n* ^a^	Median (IQR^b^)	*n* ^a^	Median (IQR^b^)	
RMS^c^ ^,*^ values	5	1.24 (1.16–2.61)	5	2.02 (0.98–2.02)	1
Distance Deviation (mm) - Lower Cutting Planes	5	0.26 (0.22–0.90)	5	1.09 (0.81–1.14)	0.310
Distance Deviation (mm) - Upper Cutting Planes	5	0.94 (0.53–1.19)	5	0.87 (0.72–1.00)	0.841
Angulation Deviation (°) - Lower Cutting Planes	5	1.86 (1.44–1.91)	5	2.42 (1.97–4.23)	0.310
Angulation Deviation (°) - Upper Cutting Planes	5	2.19 (1.84–2.74)	5	2.44 (1.73–9.97)	0.690

^a^ number of comparisons.

^b^ interquartile range.

^c^ root mean square, *comparative study (Msallem et al., 2024a).

**TABLE 4:** Comparison of the precision analysis by osteotomy techniques of the present and comparative study.

	Laser osteotomy		Conventional osteotomy		*p*-value
	*n* ^a^	Median (IQR^b^)	*n* ^a^	Median (IQR^b^)	
RMS^c^ ^,*^ values	10	1.55 (1.06–1.85)	10	1.55 (1.19–1.81)	0.912
Distance Deviation (mm) - Lower Cutting Planes	10	0.66 (0.5–1.08)	10	0.48 (0.35–0.58)	0.143
Distance Deviation (mm) - Upper Cutting Planes	10	0.48 (0.28–0.71)	10	0.78 (0.54–1.58)	0.052
Angulation Deviation (°) - Lower Cutting Planes	10	2.19 (1.03–3.47)	10	5.15 (3.09–6.03)	0.009
Angulation Deviation (°) - Upper Cutting Planes	10	2.86 (1.75–4.39)	10	8.12 (4.66–11.52)	0.009

^a^ number of comparisons.

^b^ interquartile range.

^c^ root mean square, *comparative study (Msallem et al., 2024a).

The main limitation of this study is the relatively small sample size and, thus, the likely lack of sufficient statistical power to detect minor differences. The sample size was primarily determined based on economic considerations and feasibility. Larger studies with greater sample sizes remain scarce. A study by Köhnke et al. demonstrated in a multi-center clinical study feasibility, simplicity, safety, reliability and accuracy of robot-guided laser osteotomies (Köhnke et al., 2024). Despite this, statistically significant results were achieved in favor of the laser osteotomy group in the precision analysis, which included ten comparisons. In conventional osteotomies, the reliance on cutting guides and manual handling of instruments often results in considerable deviations. The cutting guides’ planning and design are essential for optimal fit and positioning. In this study, the cutting guide for the upper osteotomy included an aiding arm for precise positioning, whereas the lower cutting guide lacked such a feature. As a result, the osteotomy procedure, including the positioning and angling of the saw and the placement of the cutting guide itself, was dependent on the operator. These factors likely contributed to deviations from the planned osteotomy cuts.

Secondary inaccuracies can also arise during the manufacturing process of the guides, such as errors during 3D printing. Fully digital workflows enable the omission of cutting guides, which enhances accuracy and reduces time and material usage (Suhaym et al., 2024). Robotic-guided laser osteotomy minimizes these challenges but is not without its limitations. Various studies have been conducted on virtual surgical planning and navigated surgery (Bernstein et al., 2017), (Han et al., 2021), (Roser et al., 2010)– (Stopp et al., 2008). The cutting widths of both the saw and laser also impact accuracy. Generally, the laser has a narrower cutting width. However, in the accuracy analysis, the reference cutting line was placed at the center of the cut to best represent the pre-planned cutting path. Nonetheless, variations in material compared to bone structure may affect the actual cutting width and the accuracy analysis. In view of the small extent, however, this was considered negligible.

While directly implementing a digitally planned osteotomy in the operating room offers several advantages, challenges can arise during the transfer of data between different software and from the digital plan to the physical model. Minor errors can accumulate during various process stages, including data transfer between software systems and registration processes. In the latter, inaccuracies may arise due to manually positioned landmarks and pointers of different shapes (Augello et al., 2018), (Ebeling et al., 2023), (Burgner et al., 2010). Additionally, the registration process and robot installation are time-consuming, leading to longer operation durations. However, studies have shown that this setup only takes a few minutes, which can be considered minimal given the possible increased efficiency of osteotomy and subsequent reconstruction (Augello et al., 2018), (Ureel et al., 2021), (Köhnke et al., 2024).

Several studies have been conducted on the feasibility of the same robot-guided laser osteotome (Holzinger et al., 2021), (Ebeling et al., 2023), (Ureel et al., 2021), (Köhnke et al., 2024). It is evident that the implementation of this technology is still in its early stages, requiring time and effort to become a standard procedure in the operating room. Nevertheless, various studies demonstrate its applicability and feasibility, with some hospitals already using it for certain standard procedures. While further pre-clinical and clinical studies are necessary to confirm the advantages, it is already feasible to integrate this system into practice.

While the initially high costs may not justify using a robotic system in many cases, including additional enhancements, such as the induction of laser-induced bone healing or the intraoperative three-dimensional repositioning of bone with a robotic system in complex reconstructions, makes them a compelling option in some instances.

Unlike previous studies that analyzed endpoints such as reconstruction with patient-specific implants, this study focused exclusively on the accuracy of the osteotomy. Only the accuracy of the cutting planes is analyzed, which does not necessarily guarantee an accurate reconstruction. This is because the placement of the screw holes with screws is also crucial and can lead to misalignment (Msallem et al., 2024a).

The laser osteotomy offers clear advantages in biological and functional terms, and the incorporation of digitization and robotics further improves accuracy and reliability, as supported by existing literature (Augello et al., 2018), (Holzinger et al., 2021), (Baek et al., 2021), (Godzik et al., 2019)– (Msallem et al., 2024a), (de Boutray et al., 2024), (Wojcik et al., 2023). However, developing standardized testing methods, particularly for surgical applications, remains an ongoing objective. Advances in digital measurement techniques will be essential for establishing these standards and improving clinical outcomes in future surgical procedures. The next step is to further underline the superiority of these systems and demonstrate their advantages in both technical and functional aspects, particularly for reconstructions and various osteotomy indications in the cranio-maxillofacial area. But additionally, expanding the applicability of these systems to other medical fields, for example, trauma and orthopedics, is highly beneficial, as many areas of medicine would greatly benefit from the precise implementation of such osteotomies.

## 5 Conclusion

This study demonstrates that robot-guided laser osteotomy provides superior accuracy compared to conventional cutting guides currently available. The findings are supported by statistically significant differences observed in the precision analyses. For the upper cutting plane, the distance deviation precision analysis revealed a statistically significant deviation of 0.48 mm in the laser osteotomy group compared to 0.78 mm the conventional osteotomy group. Similarly, the laser osteotomy group achieved statistically significant results in the angulation deviation precision analysis. For the lower cutting plane, the angulation deviation was 2.19° in the laser osteotomy group compared to 5.15° in the conventional osteotomy group. In contrast, for the upper cutting plane, the laser osteotomy group demonstrated a deviation of 2.86° and 8.12° for the conventional osteotomy group. These results highlight the enhanced accuracy of the robot-guided laser osteotomy technique, establishing it as a superior alternative to conventional techniques.

The previously conducted comparative study, which evaluated the accuracy of the reconstruction after robot-guided laser osteotomy, demonstrated that the RMS values of the two osteotomy groups exhibited comparable outcomes, rather than superiority, concerning final reconstruction. This finding underscores that current technologies have achieved clinically significant accuracy in reconstruction, with minor discrepancies becoming less impactful within the broader surgical workflow. It also suggests that different techniques may have other applications and retain their justification for specific use areas. Further studies are required to demonstrate the applicability of this innovative system in oncologic and reconstructive surgery. However, its potential to become a valuable addition to surgical procedures is promising. Beyond its ability to cut tissues with high accuracy, the system offers advanced capabilities that could enhance surgical workflows. While the high acquisition costs may limit its initial adoption, these additional functionalities could justify its implementation in the future, particularly for complex surgical applications with a high demand for accuracy.

## Abbrevations

3D, Three-dimensional; CT, Computed tomography; Er:YAG, Erbium-doped Yttrium Aluminium Garnet; IQR, Interquartile range; RMS, Root mean square; STL, Standard Tessellation Language.

## Data Availability

The original contributions presented in the study are included in the article/supplementary material, further inquiries can be directed to the corresponding author.
